# Supplementary figures and images for: COVID-19 in-hospital mortality and mode of death in a dynamic and non-restricted tertiary care model in Germany
Source: PLoS One. 2020 Nov 12;15(11):e0242127. doi: 10.1371/journal.pone.0242127 (PMC7660518; doi:10.1371/journal.pone.0242127)

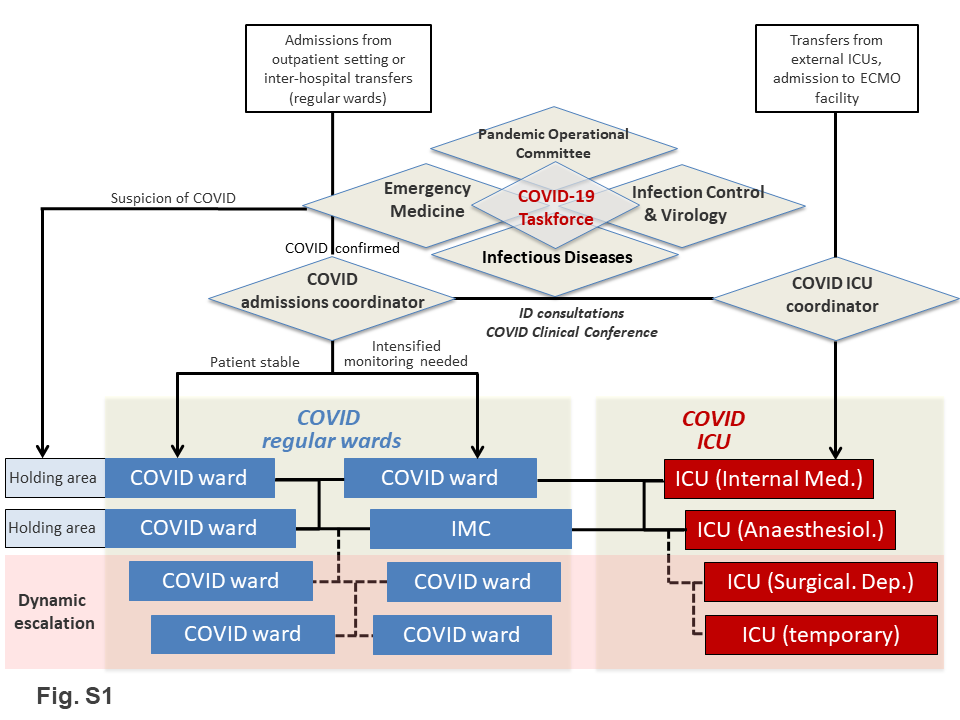

Supplement: S1 Fig — Patient flow in the dynamic care model established by the Task force Coronavirus (consisting of representatives of the ID department, Emergency department, Virology and Infection control Departments and the Pandemic Operational Committee of the University Medical Center Freiburg): Patients from the outpatient setting or inter-hospital tranfers were evaluated in dedicated areas in the emergency department. Confirmed COVID patients were distributed according to severity of disease on regular wards with or without monitoring. Patients with suspicion of COVID were admitted to separate holding areas. Unstable patients, ICU transfers or admissions to the ECMO facility were managed via the ICU coordinator and allocated to dedicated ICU and ECMO facilities. The dynamic care model included an escalation strategy, in which additional regular wards and ICU beds were equipped, physicians and nursing staff were trained and these wards were subsequently recruited upon utilisation of a certain threshold of COVID bed capacities. ID Infectious diseases, ICU Intensive care unit, IMC Intermediate care ward, COVID Coronavirus Disease 2019. (PNG) [file pone.0242127.s001.PNG]

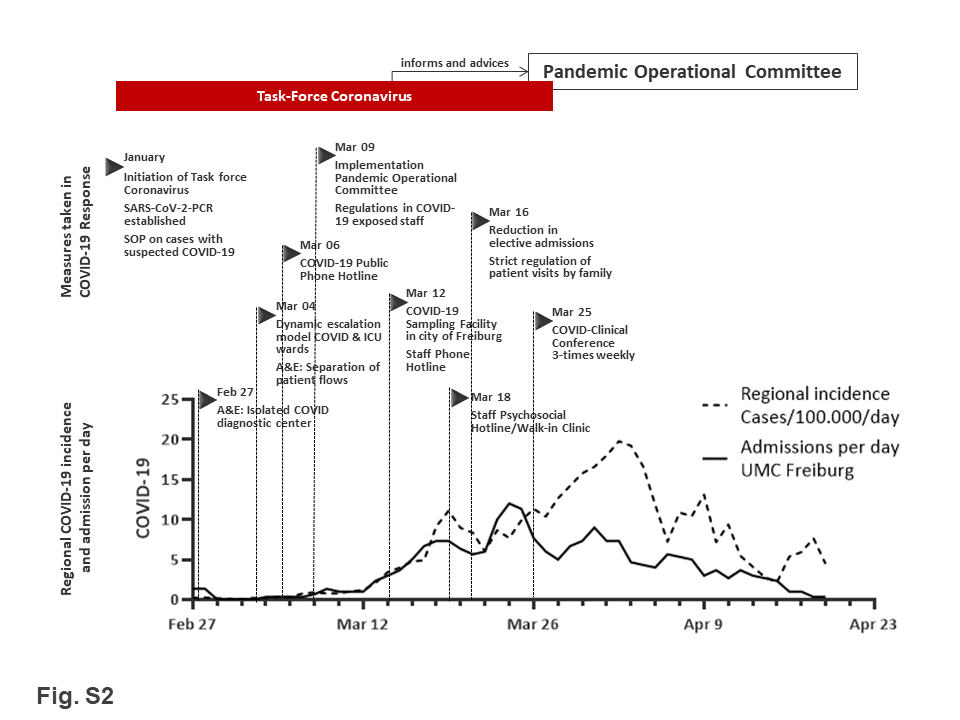

Supplement: S2 Fig — The measures implemented in the COVID-19 response, the evolution of the peak incidences in the region (COVID-19 cases/100.00/day [dates of registration at local health authorities]) and the corresponding number of admissions in the University Medical Center Freiburg. (PNG) [file pone.0242127.s002.PNG]

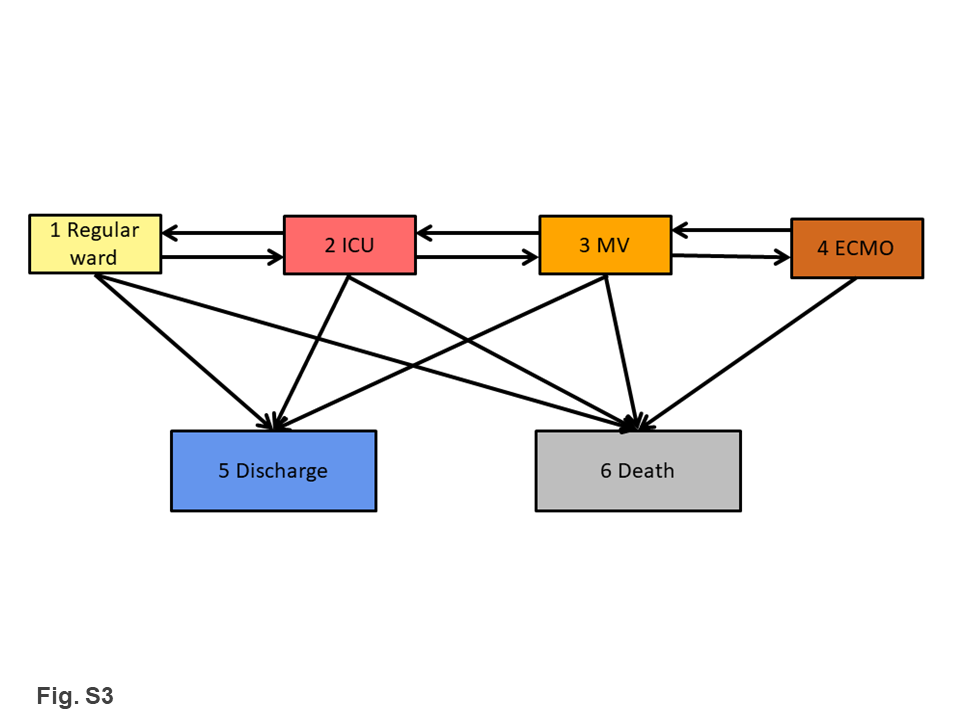

Supplement: S3 Fig — The six state model considers the events hospitalisation in 1) regular ward, 2) ICU, 3) mechanical ventilation (MV), 4) ECMO, 5) discharge and 6) death. The boxes represent the possible states a patient may encounter and the arrows represent the possible transitions from one state to another. Thus, the arrows between the states show which transitions are possible. (PNG) [file pone.0242127.s003.PNG]

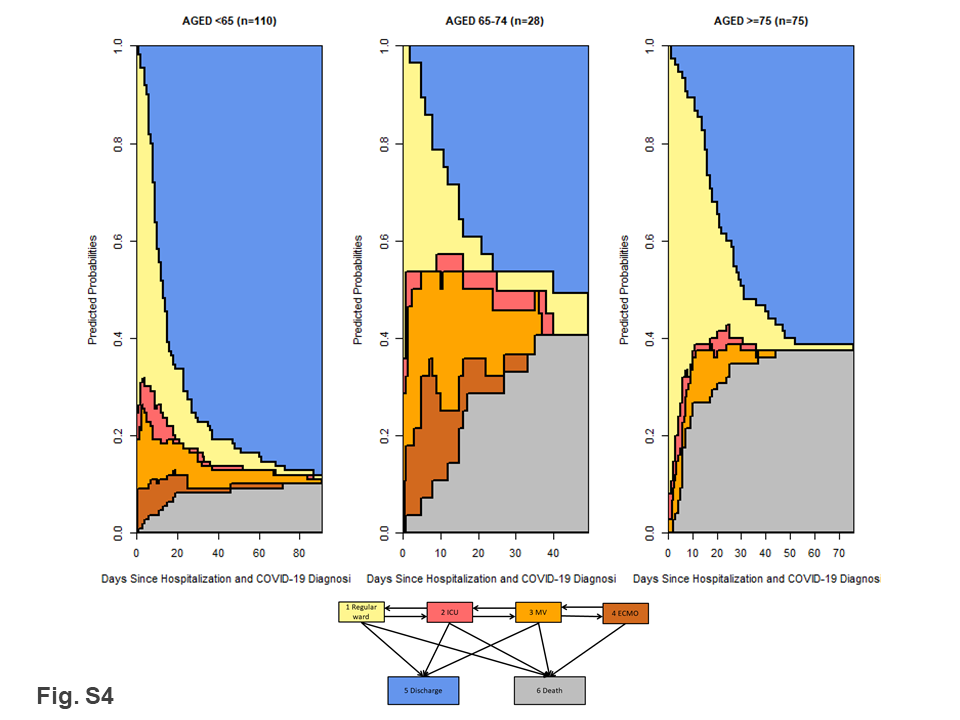

Supplement: S4 Fig — Stacked probability plots for the multistate model stratified by age. The plots illustrate in more detail the results of the competing risks regression models (however, not adjusted for other covariates). The graphs indicates that older patients have an increased risk to stay longer in hospital, to be admitted to the ICU, to need mechanical ventilation (including for a longer duration), and to die in hospital. (PNG) [file pone.0242127.s004.PNG]

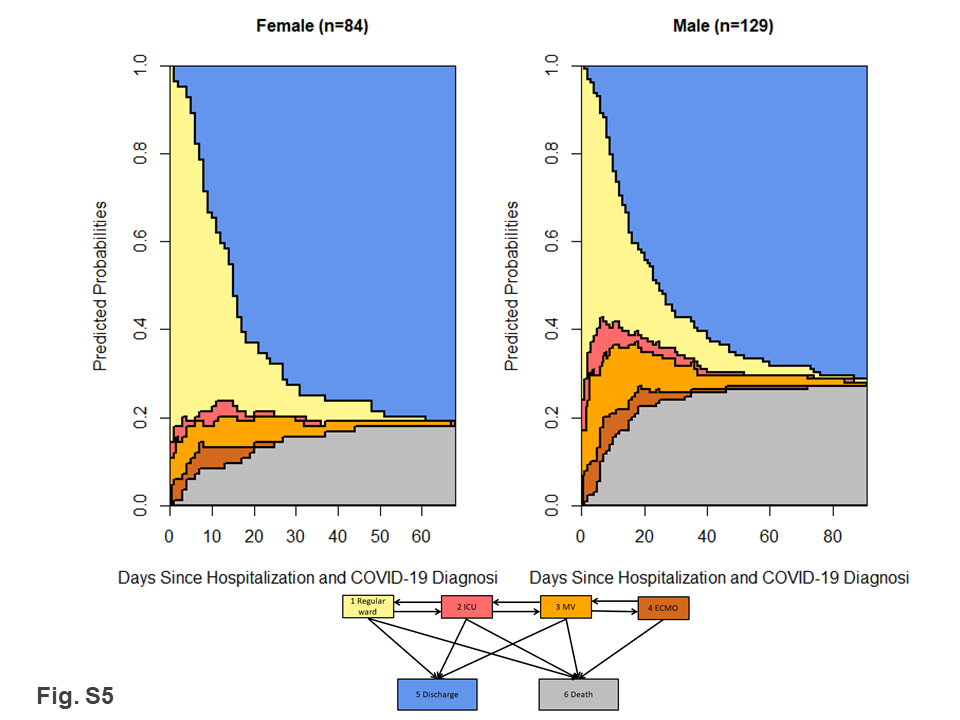

Supplement: S5 Fig — Stacked probability plots for the multistate model stratified by sex. The plots illustrate in more detail the results of the competing risks regression models (however, not adjusted for other covariates). The graphs indicates that male patients have an increased risk to be admitted to the ICU, to need mechanical ventilation, and to die in hospital. (PNG) [file pone.0242127.s005.PNG]

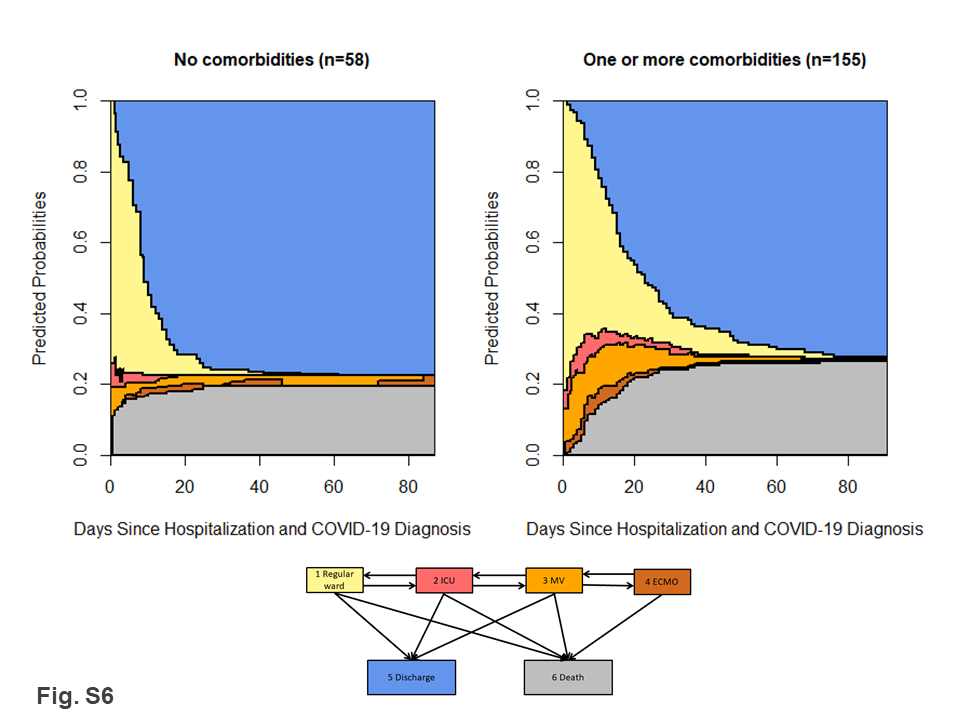

Supplement: S6 Fig — Stacked probability plots for the multistate model stratified by the presence of comorbidities. The plots illustrate in more detail the results of the competing risks regression models (however, not adjusted for other covariates). The graphs indicates that patients with one or more comorbidities have an increased risk to stay longer in hospital, to be admitted to the ICU, to need mechanical ventilation, and to die in hospital. (PNG) [file pone.0242127.s006.PNG]

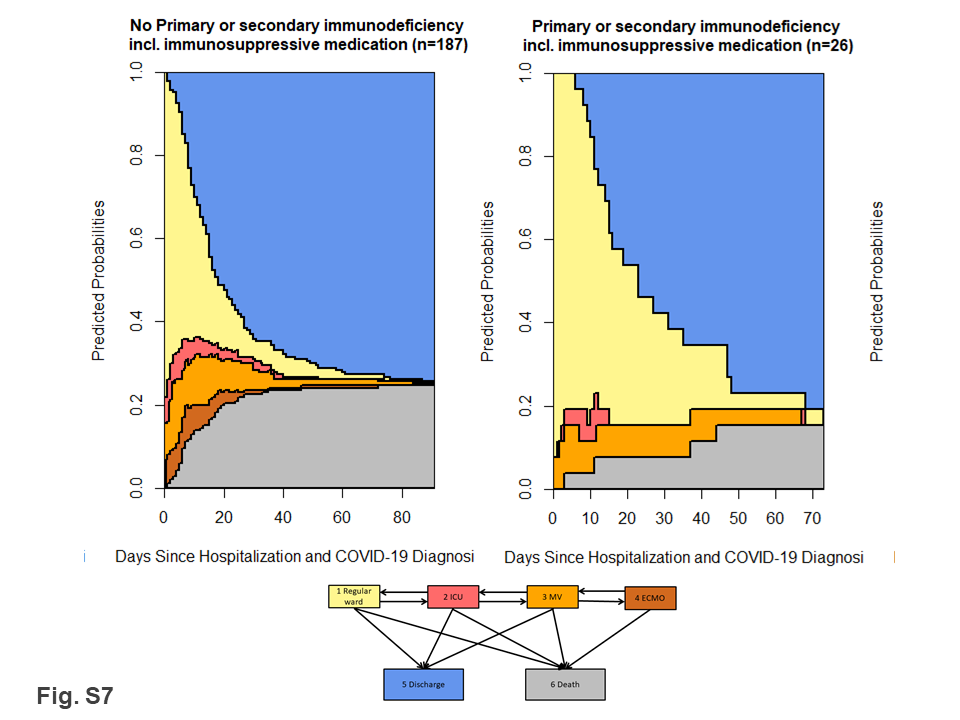

Supplement: S7 Fig — Stacked probability plots for the multistate model stratified by the presence of immunodeficiency. The plots illustrate in more detail the results of the competing risks regression models (however, not adjusted for other covariates). The graphs indicates that immunodeficient patients have an increased risk to stay longer in hospital, yet, a decreased risk to be admitted to the ICU, to need mechanical ventilation, and to die in hospital. (PNG) [file pone.0242127.s007.PNG]

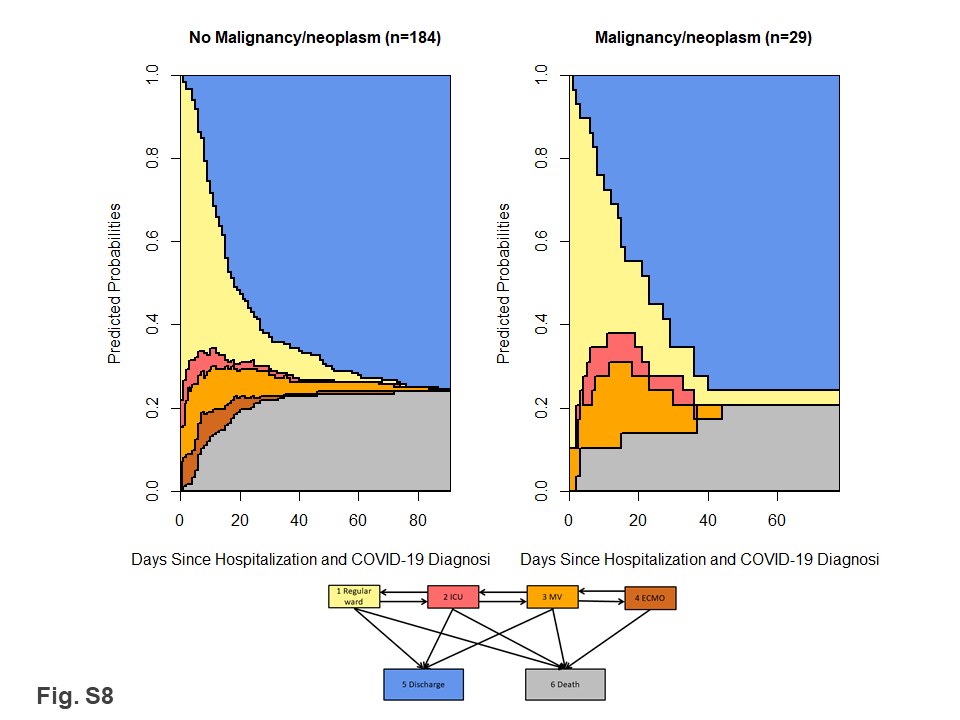

Supplement: S8 Fig — Stacked probability plots for the multistate model stratified by presence of malignancy/neoplasm. The plots illustrate in more detail the results of the competing risks regression models (however, not adjusted for other covariates). The graphs indicates that patients with malignancies or neoplasms have a slightly increased risk to be admitted to the ICU and to need mechanical ventilation, yet, no increased risk to die in hospital. (PNG) [file pone.0242127.s008.PNG]
