# Supplementary material for: COVID-19 in-hospital mortality and mode of death in a dynamic and non-restricted tertiary care model in Germany
Source: PLoS One. 2020 Nov 12;15(11):e0242127. doi: 10.1371/journal.pone.0242127 (PMC7660518; doi:10.1371/journal.pone.0242127)
Supplement: S1 Data — (HTML) [file pone.0242127.s009.html]

Supplementary Material: R code of data analysis


# Supplementary Material: R code of data analysis

#### Dr. Maja von Cube

#### 6/10/2020

```
knitr::opts_chunk$set(echo = TRUE, warning=FALSE)

# Load required packages
library(knitr)
library(mstate)
```

```
## Loading required package: survival
```

```
library(sjPlot)
library(dplyr)
```

```
## 
## Attaching package: 'dplyr'
```

```
## The following objects are masked from 'package:stats':
## 
##     filter, lag
```

```
## The following objects are masked from 'package:base':
## 
##     intersect, setdiff, setequal, union
```

```
require(png)
```

```
## Loading required package: png
```

```
source("C:/sync/Weitere Projekte/Corona Virus/Tasks/SIR3/R code DH/ext_mstate.R")
# function for preparing data set for mstate package. This function is available in 
# the online supplementary material of
# Hazard, D., Kaier, K., von Cube, M., Grodd, M., Bugiera, L., Lambert, J., & Wolkewitz, M. (2020). Joint analysis of duration of ventilation, length of intensive care, and mortality of COVID-19 patients: a multistate approach

# Load the data
my.data<-read.csv("C:/sync/Weitere Projekte/Corona Virus/Tasks/data UKL FR/Covid19_UKL_6stateJune19.csv", header=TRUE)
my.data$X<-NULL

# Data preparation-------------------------------------------------------------

# state 1: Hospital
# state 2: ICU
# state 3: MV
# state 4: ECMO
# state 5: Discharge
# state 6: Death

# Set transition matrix for mstate
tra <- transMat(x = list(c(2,5,6), c(1,3,5,6), c(2,4,5,6),c(3,6), c(),c()),
                names = c("Hospital","ICU", "MV","ECMO", "Discharge","Death"))


# Add transition vector
my.data$trans[my.data$from == 1 & my.data$to ==2] <- 1
my.data$trans[my.data$from == 1 & my.data$to ==5] <- 2
my.data$trans[my.data$from == 1 & my.data$to ==6] <- 3

my.data$trans[my.data$from == 2 & my.data$to ==1] <- 4
my.data$trans[my.data$from == 2 & my.data$to ==3] <- 5
my.data$trans[my.data$from == 2 & my.data$to ==5] <- 6
my.data$trans[my.data$from == 2 & my.data$to ==6] <- 7


my.data$trans[my.data$from == 3 & my.data$to ==2] <- 8
my.data$trans[my.data$from == 3 & my.data$to ==4] <- 9
my.data$trans[my.data$from == 3 & my.data$to ==5] <- 10
my.data$trans[my.data$from == 3 & my.data$to ==6] <- 11

my.data$trans[my.data$from == 4 & my.data$to ==3] <- 12
#my.data$trans[my.data$from == 4 & my.data$to ==5] <- 13
my.data$trans[my.data$from == 4 & my.data$to ==6] <- 13

table(my.data$trans)
```

```
## 
##   1   2   3   4   5   6   7   8   9  10  11  12  13 
##  27 149  19  26  26   8   7  28  21   4  16  12   9
```

```
# Add status vector, indicates observed transition
my.data$status <- 1

# Status vector for censored observations set to '0'
my.data$status[my.data$to == "cens"] <- 0

# Create data frame with all possible transitions for when a patient is at risk
my.data_ext <- ext_mstate(my.data, tra)

# Analysis ------------------------------------------------------------------------

## Cox model stratified by transition
c_1 <- coxph(Surv(entry, exit, status) ~ strata(trans), data= my.data_ext, method = "breslow")

# msfit of the mstate package calculates baseline hazards
msf_1 <- msfit(c_1, trans = tra)

# probtrans of the mstate package calculates transition probabilities, prediciton from day 0
pt_1 <- probtrans(msf_1, predt = 0)

# For these analysis see also 
# de Wreede, L. C., Fiocco, M., & Putter, H. (2011). mstate: an R package for the analysis of competing risks and multi-state models. Journal of statistical software, 38(7), 1-30.


# ELOS of the mstate package provides expected length of stay stratified by starting states at day 0
LOS_mat_1 <- ELOS(pt_1)
rownames(LOS_mat_1) <- c("from Hospital", "from ICU", "from MV","from ECMO", "from Discharge", "from Death")
colnames(LOS_mat_1) <- c("to Hospital", "to ICU", "to MV","to ECMO" ,"to Discharge", "to Death")
print(LOS_mat_1)
```

```
##                to Hospital    to ICU     to MV    to ECMO to Discharge to Death
## from Hospital    13.626610 0.8402273  1.354944  0.1564457     62.08356 12.93821
## from ICU          4.999015 5.5821258 13.946283  2.0443023     30.12821 34.30006
## from MV           3.951227 2.3766403 15.558197  8.0442775     21.81470 39.25495
## from ECMO         2.922306 1.5399810 10.997979 14.9078994     14.70833 45.92351
## from Discharge    0.000000 0.0000000  0.000000  0.0000000     91.00000  0.00000
## from Death        0.000000 0.0000000  0.000000  0.0000000      0.00000 91.00000
```

```
# Initial Distribution at day 0

# proportion of patients starting in state 1
prop_1<-sum(my.data[!duplicated(my.data$id),]$start_state==1)/
  n_distinct(my.data$id)
# proportion of patients starting in state 2
prop_2<-sum(my.data[!duplicated(my.data$id),]$start_state==2)/
  n_distinct(my.data$id)
# proportion of patients starting in state 3
prop_3<-sum(my.data[!duplicated(my.data$id),]$start_state==3)/
  n_distinct(my.data$id)

init_dis_1 <- c(prop_1,prop_2, prop_3, 0, 0,0)

# Multiply LOS_mat_1 with initial distribution to get weighted average of expected lengths of stay for entire cohort
LOS_cohort_1 <- (init_dis_1 %*% LOS_mat_1)
print(LOS_cohort_1)
```

```
##      to Hospital   to ICU    to MV  to ECMO to Discharge to Death
## [1,]    11.73239 1.330986 4.131455 1.410798     54.42254 17.97183
```

```
# Create weighted average of transition probabilities for progress of entire cohort
# Used to produce full cohort results.
pt_fc_1 <- pt_1
fc_1 <- pt_fc_1[[1]] * prop_1 + pt_fc_1[[2]] * prop_2 +pt_fc_1[[3]] * prop_3
pt_fc_1[[1]] <- fc_1

dim_pt<-dim(pt_fc_1[[1]])[1]
```

```
img1_path <- "C:/sync/Weitere Projekte/Corona Virus/Tasks/data UKL FR/figures/6state_model_color.png"
img1 <- readPNG(img1_path, native = TRUE, info = TRUE)
include_graphics(img1_path)
```

```
img2_path <- "C:/sync/Weitere Projekte/Corona Virus/Tasks/data UKL FR/figures/cohortplot_sorted.png"
img2 <- readPNG(img1_path, native = TRUE, info = TRUE)
include_graphics(img2_path)
```

The five state model considers the events hospitalization (in reuglar ward), ICU, mechanical ventilation (MV), ECMO, discharge and death. The Figure shows which transitions are possible. For example, all patients with ECMO must receive MV without ECMO before being in the ICU with neither ECMO nor MV. Moreover, a patient cannot be discharged from the ICU directly from the MV state. If a patient moves from ECMO to ICU without being MV in between, we artificially add 0.5 days of MV. If a patient moves from MV directly to a normal ward, we artificially add 0.5 days of ICU stay. As time scale, we use time since hospitalization with COVID-19 diagnosis.

We have 12 direct admissions to the ICU and 31 to the ICU with MV. In total, 70 patients are admitted to the ICU, 57 need MV and 23 MV and ECMO.

```
par(mfrow=c(1,1))
plot(pt_fc_1, from = 1,  ord = c(6,4,3,2,1,5),type= "filled",
     cols = c("khaki1","indianred1", "orange","chocolate", "cornflowerblue","gray"),
     lwd= 2, xlab = "Days Since Hospitalization with COVID-19 Diagnosis", ylab = "Predicted Probabilities", 
     cex.lab = 1, legend = c("", "", "", "","","") ,    main= "COVID-19 Patients 
(Medical Center Freiburg)", cex.main=1)
text(10, 0.5, "Hospital", cex = 1)
text(12, 0.35, "ICU", cex = 1)
text(15, 0.27, "MV", cex = 1)
text(15, 0.2, "ECMO", cex = 1)
text(50, 0.9, "Discharge", cex = 1)
text(50, 0.1, "Death", cex = 1)
```

```
par(mfrow=c(1,3))
plot(pt_1, from = 1,  ord = c(6,4,3,2,1,5),type= "filled",
     cols = c("khaki1","indianred1", "orange","chocolate", "cornflowerblue","gray"),
     lwd= 2, xlab = "DDays Since Hospitalization with COVID-19 Diagnosis", ylab = "Predicted Probabilities", 
     cex.lab = 1, legend = c("", "", "", "","","") ,    main= "COVID-19 Patients 
(Medical Center Freiburg)", cex.main=1)
text(15, 0.4, "Hospital", cex = 1)
text(13, 0.22, "ICU", cex = 1)
text(22, 0.19, "MV", cex = 1)
text(20, 0.16, "ECMO", cex = 1)
text(50, 0.9, "Discharge", cex = 1)
text(50, 0.1, "Death", cex = 1)  

plot(pt_1, from = 2, ord = c(6,4,3,2,1,5),type= "filled",
     cols = c("khaki1","indianred1", "orange","chocolate", "cornflowerblue","gray"),
     lwd= 2, xlab = "Days Since Hospitalization with COVID-19 Diagnosis", ylab = "Predicted Probabilities", 
     cex.lab = 1, legend = c("", "", "", "","","") ,    main= "COVID-19 Patients 
(Medical Center Freiburg)", cex.main=1)
text(56, 0.61, "Hospital", cex = 1)
text(5, 0.85, "ICU", cex = 1)
text(20, 0.6, "MV", cex = 1)
text(18, 0.4, "ECMO", cex = 1)
text(50, 0.9, "Discharge", cex = 1)
text(50, 0.1, "Death", cex = 1)  

plot(pt_1, from = 3, ord = c(6,4,3,2,1,5),type= "filled",
     cols = c("khaki1","indianred1", "orange","chocolate", "cornflowerblue","gray"),
     lwd= 2, xlab = "Days Since Hospitalization with COVID-19 Diagnosis", ylab = "Predicted Probabilities", 
     cex.lab = 1, legend = c("", "", "", "","","") ,    main= "COVID-19 Patients 
(Medical Center Freiburg)", cex.main=1)
text(56, 0.67, "Hospital", cex = 1)
text(55, 0.63, "ICU", cex = 1)
text(20, 0.5, "ECMO", cex = 1)
text(20, 0.7, "MV", cex = 1)
text(50, 0.9, "Discharge", cex = 1)
text(50, 0.1, "Death", cex = 1)
```

#### Lengths of stay, duration of MV, risk of death and discharge (population based; 5-state-model)

```
LOS_cohort_1 <- (init_dis_1 %*% LOS_mat_1)
print(LOS_cohort_1)
```

```
##      to Hospital   to ICU    to MV  to ECMO to Discharge to Death
## [1,]    11.73239 1.330986 4.131455 1.410798     54.42254 17.97183
```

```
print(pt_fc_1[[1]][dim_pt,1:7])
```

```
##    time     pstate1 pstate2 pstate3 pstate4   pstate5   pstate6
## 70   91 0.004694836       0       0       0 0.7558685 0.2394366
```

#### Lengths of stay, duration of MV, risk of death and discharge (Individual based; 5-state model)

```
print(LOS_mat_1)
```

```
##                to Hospital    to ICU     to MV    to ECMO to Discharge to Death
## from Hospital    13.626610 0.8402273  1.354944  0.1564457     62.08356 12.93821
## from ICU          4.999015 5.5821258 13.946283  2.0443023     30.12821 34.30006
## from MV           3.951227 2.3766403 15.558197  8.0442775     21.81470 39.25495
## from ECMO         2.922306 1.5399810 10.997979 14.9078994     14.70833 45.92351
## from Discharge    0.000000 0.0000000  0.000000  0.0000000     91.00000  0.00000
## from Death        0.000000 0.0000000  0.000000  0.0000000      0.00000 91.00000
```

```
print("Starting in 1 (hospital)")
```

```
## [1] "Starting in 1 (hospital)"
```

```
print(pt_1[[1]][dim_pt,1:7])
```

```
##    time     pstate1 pstate2 pstate3 pstate4   pstate5   pstate6
## 70   91 0.003571122       0       0       0 0.8331677 0.1632612
```

```
print("Starting in 2 (ICU)")
```

```
## [1] "Starting in 2 (ICU)"
```

```
print(pt_1[[2]][dim_pt,1:7])
```

```
##    time     pstate1 pstate2 pstate3 pstate4   pstate5   pstate6
## 70   91 0.008576868       0       0       0 0.5179798 0.4734434
```

```
print("Starting in 3 (MV)")
```

```
## [1] "Starting in 3 (MV)"
```

```
print(pt_1[[3]][dim_pt,1:7])
```

```
##    time     pstate1 pstate2 pstate3 pstate4   pstate5   pstate6
## 70   91 0.009354416       0       0       0 0.4240558 0.5665898
```

# Cox Regression for competing risks model

```
img1_path <- "C:/sync/Weitere Projekte/Corona Virus/Tasks/data UKL FR/figures/3state_model.png"
img1 <- readPNG(img1_path, native = TRUE, info = TRUE)
include_graphics(img1_path)
```

For the regression analysis, we employ a competing risks model with endpoints discharge alive from hospital and death in hospital.

First, we perform cause-specific Cox regression. The hazard ratio indicates to what extend the event rate is increased/decreased by a specific risk factor compared to the baseline rate.

Then, we estimate subdistribution hazard ratios. The subdistribution hazard ratio indicates to what extend the risk of an event is increased/decreased by a specific risk factor compared to the baseline risk. In this setting, the proportionality assumption is likely violated.

```
# load the data  
my.data<-read.csv("C:/sync/Weitere Projekte/Corona Virus/Tasks/data UKL FR/Covid19_UKL_6stateJune19.csv", header=TRUE)
my.data$X<-NULL

# data preparation
# One line per patient with final outcome (discharge, death or censored)
my.data<-my.data[my.data$to%in%c("5", "6", "cens"),]
my.data$from<-0
my.data$entry<-0
#my.data$to<-droplevels(my.data$to)

# create age categories
my.data$age_cat<-0
my.data$age_cat[my.data$Age>65 & my.data$Age<=74]<-2
my.data$age_cat[my.data$Age>=75]<-3
my.data$age_cat<-as.factor(my.data$age_cat)
my.data$age_cat<-relevel(my.data$age_cat, ref="0")

# create co-morbidity categories
my.data$com_cat<-0
my.data$com_cat[my.data$comorbidity_mitBer>=1]<-1
```

### Summary of the covariates

```
print("Sex")
```

```
## [1] "Sex"
```

```
table(my.data$Sex)
```

```
## 
##   f   m 
##  84 129
```

```
print("Age (in categories  0: 0-64 ; 1:65-74 ; 2: >=75")
```

```
## [1] "Age (in categories  0: 0-64 ; 1:65-74 ; 2: >=75"
```

```
table(my.data$age_cat)
```

```
## 
##   0   2   3 
## 110  28  75
```

```
print("Reference level is Category 0")
```

```
## [1] "Reference level is Category 0"
```

```
print("Erwerb_Infek (hospital versus community acquired COVID-19 infection")
```

```
## [1] "Erwerb_Infek (hospital versus community acquired COVID-19 infection"
```

```
table(my.data$Erwerb_Infek)
```

```
## 
##   ambulant nosokomial 
##        163         50
```

```
print("comorbidity_mitBer: Comorbidities")
```

```
## [1] "comorbidity_mitBer: Comorbidities"
```

```
table(my.data$comorbidity_mitBer)
```

```
## 
##  0  1  2  3  4 
## 58 80 54 15  6
```

```
table(my.data$com_cat)
```

```
## 
##   0   1 
##  58 155
```

```
print("LOSdummy: Length of hospital stay prior to COVID-19 acquistion (0 for community acquired COVID-19)")
```

```
## [1] "LOSdummy: Length of hospital stay prior to COVID-19 acquistion (0 for community acquired COVID-19)"
```

```
summary(my.data$LOSdummy)
```

```
##    Min. 1st Qu.  Median    Mean 3rd Qu.    Max. 
##   0.000   0.000   0.000   3.296   1.000 185.000
```

### Multivariable cause-specific Cox regression (hazard ratio)

```
tab_model(coxph(Surv(entry, exit, to==5) ~ Sex+ factor(age_cat)  + Erwerb_Infek + 
                  factor(com_cat)+LOSdummy , data= my.data, method = "breslow"), title="Discharge")
```

Discharge

|  | Dependent variable | | |
| Predictors | Estimates | CI | p |
| Sex [m] | 0.68 | 0.50 – 0.94 | **0.020** |
| age\_cat [2] | 0.63 | 0.37 – 1.06 | 0.079 |
| age\_cat [3] | 0.71 | 0.49 – 1.02 | 0.067 |
| Erwerb\_Infek [nosokomial] | 0.73 | 0.48 – 1.12 | 0.155 |
| com\_cat [1] | 0.87 | 0.60 – 1.25 | 0.442 |
| LOSdummy | 1.00 | 0.99 – 1.01 | 0.739 |
| Observations | 213 | | |
| R2 Nagelkerke | 0.105 | | |

```
tab_model(coxph(Surv(entry, exit, to==6) ~ Sex+ factor(age_cat)  +  Erwerb_Infek + 
                  factor(com_cat)+LOSdummy , data= my.data, method = "breslow"),
          title="Death")
```

Death

|  | Dependent variable | | |
| Predictors | Estimates | CI | p |
| Sex [m] | 1.37 | 0.74 – 2.54 | 0.310 |
| age\_cat [2] | 3.45 | 1.49 – 7.98 | **0.004** |
| age\_cat [3] | 3.56 | 1.74 – 7.30 | **0.001** |
| Erwerb\_Infek [nosokomial] | 0.91 | 0.45 – 1.84 | 0.790 |
| com\_cat [1] | 1.30 | 0.61 – 2.79 | 0.494 |
| LOSdummy | 0.98 | 0.94 – 1.02 | 0.372 |
| Observations | 213 | | |
| R2 Nagelkerke | 0.324 | | |

### Multivariable Subdistribution hazard ratio

```
fg_data<-crprep(Tstop="exit", status="to", data=my.data, trans=c("5", "6"), cens="cens", 
             Tstart="entry",id="id",
       keep=c("Sex", "age_cat", "com_cat", "LOSdummy","Erwerb_Infek" ))


tab_model(coxph(Surv(Tstart,Tstop,status=="5")~Sex+ factor(age_cat)  +  Erwerb_Infek + 
                factor(com_cat)+LOSdummy, data=fg_data,
              weight=weight.cens, subset=failcode=="5"),
          title="Fine and gray model: Discharge", show.p = FALSE)
```

Fine and gray model: Discharge

|  | Dependent variable | |
| Predictors | Estimates | CI |
| Sex [m] | 0.66 | 0.48 – 0.90 |
| age\_cat [2] | 0.44 | 0.26 – 0.74 |
| age\_cat [3] | 0.46 | 0.32 – 0.66 |
| Erwerb\_Infek [nosokomial] | 0.80 | 0.52 – 1.23 |
| com\_cat [1] | 0.79 | 0.55 – 1.13 |
| LOSdummy | 1.00 | 0.99 – 1.01 |
| Observations | 313 | |
| R2 Nagelkerke | 0.210 | |

```
cox<-summary(coxph(Surv(Tstart,Tstop,status=="5")~Sex+ factor(age_cat)  +  Erwerb_Infek + 
                factor(com_cat)+LOSdummy, data=fg_data,
              weight=weight.cens, subset=failcode=="5"))


tab_model(coxph(Surv(Tstart,Tstop,status=="6")~Sex+ factor(age_cat)  +  Erwerb_Infek + 
                factor(com_cat)+LOSdummy, data=fg_data,
              weight=weight.cens, subset=failcode=="6"),
          title="Fine and gray model: Death", show.p = FALSE)
```

Fine and gray model: Death

|  | Dependent variable | |
| Predictors | Estimates | CI |
| Sex [m] | 1.90 | 1.04 – 3.48 |
| age\_cat [2] | 4.16 | 1.82 – 9.49 |
| age\_cat [3] | 4.13 | 2.05 – 8.32 |
| Erwerb\_Infek [nosokomial] | 1.18 | 0.60 – 2.34 |
| com\_cat [1] | 1.25 | 0.59 – 2.68 |
| LOSdummy | 0.98 | 0.94 – 1.03 |
| Observations | 531 | |
| R2 Nagelkerke | 0.426 | |

### p-values Subdistribution hazard ratio

```
fg_data<-crprep(Tstop="exit", status="to", data=my.data, trans=c("5", "6"), cens="cens", 
             Tstart="entry",id="id",
       keep=c("Sex", "age_cat", "com_cat", "LOSdummy","Erwerb_Infek" ))


summary(coxph(Surv(Tstart,Tstop,status=="5")~Sex+ factor(age_cat)  +  Erwerb_Infek + 
                factor(com_cat)+LOSdummy, data=fg_data,
              weight=weight.cens, subset=failcode=="5"))
```

```
## Call:
## coxph(formula = Surv(Tstart, Tstop, status == "5") ~ Sex + factor(age_cat) + 
##     Erwerb_Infek + factor(com_cat) + LOSdummy, data = fg_data, 
##     weights = weight.cens, subset = failcode == "5")
## 
##   n= 313, number of events= 161 
## 
##                             coef exp(coef)  se(coef) robust se      z Pr(>|z|)
## Sexm                   -0.422271  0.655557  0.161082  0.153628 -2.749  0.00598
## factor(age_cat)2       -0.817358  0.441597  0.265097  0.256761 -3.183  0.00146
## factor(age_cat)3       -0.775018  0.460696  0.187259  0.166305 -4.660 3.16e-06
## Erwerb_Infeknosokomial -0.218936  0.803373  0.217695  0.195464 -1.120  0.26268
## factor(com_cat)1       -0.238165  0.788073  0.183121  0.177481 -1.342  0.17962
## LOSdummy               -0.000262  0.999738  0.005058  0.001968 -0.133  0.89409
##                           
## Sexm                   ** 
## factor(age_cat)2       ** 
## factor(age_cat)3       ***
## Erwerb_Infeknosokomial    
## factor(com_cat)1          
## LOSdummy                  
## ---
## Signif. codes:  0 '***' 0.001 '**' 0.01 '*' 0.05 '.' 0.1 ' ' 1
## 
##                        exp(coef) exp(-coef) lower .95 upper .95
## Sexm                      0.6556      1.525    0.4851    0.8859
## factor(age_cat)2          0.4416      2.265    0.2670    0.7304
## factor(age_cat)3          0.4607      2.171    0.3325    0.6382
## Erwerb_Infeknosokomial    0.8034      1.245    0.5477    1.1784
## factor(com_cat)1          0.7881      1.269    0.5565    1.1159
## LOSdummy                  0.9997      1.000    0.9959    1.0036
## 
## Concordance= 0.654  (se = 0.021 )
## Likelihood ratio test= 38.01  on 6 df,   p=1e-06
## Wald test            = 45.96  on 6 df,   p=3e-08
## Score (logrank) test = 39.65  on 6 df,   p=5e-07,   Robust = 47.92  p=1e-08
## 
##   (Note: the likelihood ratio and score tests assume independence of
##      observations within a cluster, the Wald and robust score tests do not).
```

```
summary(coxph(Surv(Tstart,Tstop,status=="6")~Sex+ factor(age_cat)  +  Erwerb_Infek + 
                factor(com_cat)+LOSdummy, data=fg_data,
              weight=weight.cens, subset=failcode=="6"))
```

```
## Call:
## coxph(formula = Surv(Tstart, Tstop, status == "6") ~ Sex + factor(age_cat) + 
##     Erwerb_Infek + factor(com_cat) + LOSdummy, data = fg_data, 
##     weights = weight.cens, subset = failcode == "6")
## 
##   n= 531, number of events= 51 
## 
##                            coef exp(coef) se(coef) robust se      z Pr(>|z|)
## Sexm                    0.64126   1.89888  0.30921   0.29561  2.169 0.030063
## factor(age_cat)2        1.42490   4.15745  0.42133   0.39476  3.609 0.000307
## factor(age_cat)3        1.41900   4.13297  0.35676   0.34177  4.152  3.3e-05
## Erwerb_Infeknosokomial  0.16838   1.18339  0.34807   0.31213  0.539 0.589572
## factor(com_cat)1        0.22519   1.25255  0.38842   0.37288  0.604 0.545909
## LOSdummy               -0.01752   0.98264  0.02301   0.01469 -1.193 0.233023
##                           
## Sexm                   *  
## factor(age_cat)2       ***
## factor(age_cat)3       ***
## Erwerb_Infeknosokomial    
## factor(com_cat)1          
## LOSdummy                  
## ---
## Signif. codes:  0 '***' 0.001 '**' 0.01 '*' 0.05 '.' 0.1 ' ' 1
## 
##                        exp(coef) exp(-coef) lower .95 upper .95
## Sexm                      1.8989     0.5266    1.0638     3.389
## factor(age_cat)2          4.1575     0.2405    1.9178     9.013
## factor(age_cat)3          4.1330     0.2420    2.1152     8.076
## Erwerb_Infeknosokomial    1.1834     0.8450    0.6419     2.182
## factor(com_cat)1          1.2526     0.7984    0.6031     2.601
## LOSdummy                  0.9826     1.0177    0.9548     1.011
## 
## Concordance= 0.707  (se = 0.034 )
## Likelihood ratio test= 28.33  on 6 df,   p=8e-05
## Wald test            = 26.61  on 6 df,   p=2e-04
## Score (logrank) test = 27.11  on 6 df,   p=1e-04,   Robust = 25.02  p=3e-04
## 
##   (Note: the likelihood ratio and score tests assume independence of
##      observations within a cluster, the Wald and robust score tests do not).
```
